# Does Self-Objectification Entail an Opposition Between Appearance and Competence? The Likert Version of the Self-Objectification Questionnaire (LSOQ)

**DOI:** 10.5334/pb.481

**Published:** 2021-02-08

**Authors:** Robin Wollast, Olivier Klein, Dawn M. VanLeeuwen, Sarah J. Gervais, Philippe Bernard

**Affiliations:** 1 Université libre de Bruxelles, BE; 2 New Mexico State University, US; 3 University of Nebraska, Lincoln, US

**Keywords:** self-objectification, objectification, physical appearance, reliability, generalizability theory

## Abstract

We propose a new method to test the reliability of Fredrickson et al.’s self-objectification questionnaire (SOQ). This scale being based on a ranking, traditional reliability estimates are inappropriate. Based on generalizability theory, we suggest to compute the reliability of each subset of questions related to physical appearance vs. physical competence separately in order to average them. We applied this method to a sample of female US undergraduates (*n* = 395) and evidenced that the reliability of the scale is very low (corrected Cronbach’s alpha = .31). We also noted that a large proportion of the sample (32%) failed to complete the scale correctly. In a second study (*n* = 93), we propose a Likert adaptation of the scale and show that the two dimensions of the SOQ are independent. In Study 3 (*n* = 195), we confirm results of Study 2 and demonstrate that the general structure of the Likert version has satisfactory model fit statistics. These observations lead us to discourage the use of the original version of the SOQ and rely on the Likert version of the Self-Objectification Questionnaire (LSOQ, see appendix).

“Sexual objectification is the experience of being treated as a body (or collection of body parts) valued predominantly for its use to (or consumption by) others” write Fredrickson and Roberts (1997, p. 174). In their objectification theory, they argue that women are commonly exposed to this experience and, as a consequence, come to “self-objectify” i.e., to “treat themselves as objects to be looked at and evaluated” (p. 177). According to Fredrickson and Roberts, self-objectification can be either a state, induced by contextual cues (e.g., such as the presence of a mirror or a male gaze) or a chronic trait. In turn, self-objectification is thought to impair mental health. Eating disorders, sexual dysfunctions and depression are some of its possible consequences.

Under the inspiration of the theory formulated by these two scholars, this concept has attracted considerable research (for a review, see Moradi & Huang, 2008). To test this theory, an adequate measurement of self-objectification was necessary. The most popular one has been the Self-Objectification Questionnaire (SOQ) initially developed by Noll and Fredrickson (1998). The purpose of this paper is to provide a proper test of the internal consistency of this scale, given potential issues with reliability that have been raised by other researchers (Calogero, 2011; Hill & Fischer, 2008). Toward that end, we hope to provide reliability evidence that can equip researchers with a tool, either the SOQ or a Likert version of this scale (LSOQ), to assess self-objectification in their own research.

The use of this scale involves asking participants to rank twelve bodily attributes in terms of their importance for their physical self-concept, in ascending order from rank 1 (*most impact*) to rank 12 (*least impact*). Participants can only attribute one rank per attribute. Half the attributes are appearance related (weight, sex appeal, muscle tone, physical attractiveness, measurements [e.g., chest, waist, hips], coloring) and the other half are competency related (physical coordination, health, muscular strength, physical fitness, physical energy level, stamina). The list was later reduced to 10 attributes (from rank 0 = *least impact* to rank 9 = *greatest impact*) in an influential paper by Fredrickson et al. (1998) in which the authors combined stamina and physical energy level in one item (i.e., energy level), replaced muscular strength into strength, changed muscle tone to firm/sculpted muscles and omitted coloring. To obtain a total score, the sum of the competency items is subtracted from the sum of the appearance items (ranging from –25 to +25), with higher scores indicating greater self-objectification. This 10-item version has been most frequently used.

Since the original publication of objectification theory (Fredrickson & Roberts, 1997), this topic has garnered intense attention. For example, a recent search relying on the Google Scholar database, yields more than 5000 citations of Fredrickson and Robert’s seminal paper. Having been developed by the authors of this theory, the SOQ has been the most used measure of this construct. The evidence suggests that it is a valid measure of self-objectification: It indeed predicts a large number of outcomes consistent with objectification theory such as body image concerns and eating disorders (for a review, see Moradi & Huang, 2008).

Yet, the original creators of the scale have not reported a proper test of the reliability of the scale. One possible reason for this is that traditional reliability estimates cannot be readily used on this scale. This is because the subscales sum to a constant (VanLeeuwen & Mandabach, 2002) so that the competence score is always equal to 45 minus the appearance score and vice versa.

Researchers have recommended alternative methods of assessing reliability. Hill and Fischer (2008), for example, recommended reporting the correlation between the sums of scores on the appearance-based and competence-based items. The more negative this correlation, the higher the reliability. This recommendation was later adopted by other scholars (see e.g., Calogero, 2011; Calogero & Jost, 2011) and is the only attempt to report the reliability of this scale that we are aware of. However, given the structure of the scale, if participants correctly completed it, the correlation will always be –1. Hence, correlations that differ from this value most likely reflect participants’ failures to comply with the instructions. Thus, calculating the correlation between the sums of scores tells little about reliability of the SOQ.

In addition to implying that the correlation between the appearance and competence sums is –1, the sum to a constant constraint imposed by ranking implies that for each item, the sum of the covariance with all items in the SOQ (including the covariance of the item with itself) is zero. It also mathematically implies that the covariance between some pairs of items must be negative. But reliability theory posits that a subject’s response to an individual item reflects her true value on the underlying construct (i.e., a subject^1^ effect) and random error, and, when items are not parallel, also an item effect (Cronbach, Gleser, Nanda, & Rajaratnam, 1972). Estimated reliability is higher the larger the subject variance component is relative to the measurement error variance. Even though the self-objectification score is typically represented as the difference between the appearance and competence scores, because these scores sum to a constant (45), the self-objectification score can be represented in terms of only the appearance score or only the competence score. Consequently, reliability for the self-objectification score is equal to the reliability of the appearance or competence score and the usual notions of reliability can be applied to either the appearance or competence score. Then the self-objectification score will be reliable when the appearance (or competence) subscale subject variance is large relative to the measurement error variance. A positive subject variance implies that covariances among the appearance items (or the competence items) are positive on average, with the sum to zero constraint met by having negative covariance between pairs of items that include one item each from the appearance and competence lists.

One of the main goals of this paper is to test the reliability of the commonly used SOQ. This aim is important because having a reliable scale increases our confidence that the empirical investigations of objectification theory are valid indeed, reliability and validity go hand-in-hand. By definition, an unreliable scale (plagued by random error) cannot adequately measure what it purports to measure and hence, cannot be valid. For this reason, while the purpose of this paper is not to establish the validity of the scale per se, by considering the reliability of this scale, we indirectly address the issue of validity.

The SOQ has many strengths; it is a face valid measure that maps onto Fredrickson and Roberts’ (1997) original theorizing of the self-objectification construct. It is also unique and does not overlap with measures of closely related constructs, possibly because of its idiosyncratic approach to measuring self-objectification. The objectified body consciousness scale (OBCS), for example, was developed around the same time and describes the effect of the internalization of body standards among women (McKinley & Hyde, 1996). Although the SOQ and OBCS are closely related, the SOQ is the only one to measure the importance that one places on observable physical attributes relative to non-observable physical attributes, or whether one is literally adopting a perspective in line with a third-party observer.

Given the uniqueness and popularity of the SOQ in research, the present article is the first to propose a proper test of its internal consistency. To do so, we rely on the cohesive framework for examining reliability provided by generalizability theory to estimate scale reliability (Cronbach, Gleser, Nanda, & Rajaratnam, 1972; Shavelson & Webb, 1991; Brennan, 1992).

## General approach

Generalizability theory (Cronbach et al., 1972; Shavelson, Webb, & Rowley, 1989) defines reliability in terms of variance components. Each individual response to an item can be expressed as a function of the item, the subject and an error (i.e., interaction between the subject and the item). Thus, the variance in scores on a given item can be expressed as:

1 \sigma _S^2 + \sigma _I^2 + \sigma _{SI,e}^2

where \sigma _S^2 designates the variance due to subjects, \sigma _I^2 the variance due to items, and the error term the interaction between subjects and items. Here since subjects are the object of measurement with items representing a facet, a reliability for a relative decision (i.e., for comparing subjects) might be thought of as the subject variability/(the subject variability + the error variability of a subject comparison). The reliability of a scale can then be estimated as the proportion of the total variance that is due to participants as opposed to items or the interaction between item and errors. This can be expressed as follows using ANOVA formulation. This is the reliability for relative decisions (cf. Brennan, 1992):

2 \alpha = \frac{{M{S_S} - M{S_{Res}}}}{{M{S_s}}} = \frac{{\hat \sigma _S^2}}{{\hat \sigma _S^2 + \frac{{\hat \sigma _{SI,e}^2}}{k}}}

k represents the number of items in the scale. MS_S_ and MS_Res_ are the mean square for the subject and for the residual term, respectively. This reliability coefficient is equivalent to Cronbach’s alpha and so can also be represented as a ratio based on the average variance within items and the average covariance among items (Cronbach, et al., 1972; Brennan 1992).

As already mentioned, creating a measurement model for the SOQ poses specific problems because of the ranking and the constant sum. By construction, rankings and ratings behave differently. For example, if subjects assigned ratings to items in a completely random fashion (i.e., independently), the correlation between pairs of items will be zero. But if subjects rank items randomly, the constant sum constraint implies a negative correlation of –(1/(k–1)) between items. Ranking also forces subjects to assign different values to items they might rate the same. However, while rankings and ratings behave differently, and ranking implies that the average covariance among all the items must be negative, this is not true of subsets of the ranked items. A subject with a high SOQ score has assigned mostly high ranks to the appearance items (i.e., low ranks were assigned to the competence items) and a subject with a low SOQ score has assigned mostly low ranks to the appearance items (or conversely high ranks to the competence items). If this type of pattern prevails, then covariance values among item pairs within the appearance score (or equivalently the competence score) may be positive, reflecting a subject (or true score) effect, with the sum to zero constraint satisfied by having negative covariance between appearance-competence item pairs. If this is the case, the above measurement model, while typically applied to ratings, might also be applied to either the appearance or competence item subsets.

The forced choice imposed by requiring subjects to rank the SOQ items along with differencing the appearance and competence subscale scores is consistent with appearance and competence conceptualized as being on opposite ends of a single scale. But the SOQ score can be expressed as a function of either of the two subscales. Let us call “Appearance” and “Competence” the sum of the rankings on each of these subscales and “SOQ” the score on the SOQ.

3 {\rm SOQ}\; = \;{\rm Appearance}\; -{\rm Competence}

4 {\rm Competence}\; = \;45\; -{\rm Appearance}

Hence:

5 {\rm SOQ}\; = {\rm Appearance}-(45 -{\rm Appearance})

6 {\rm{SOQ}}\; = \;{2}\;*\;{\rm{Appearance}} -{45}

A similar sequence shows an alternative expression using the Competence score:

7 {\rm SOQ}\; = 45-2 *{\rm Competence}

It follows that the reliability of the whole scale can be estimated by examining the reliability of either subscale. Because both subscales provide a reliability estimate using only part of the data, we suggest averaging the reliabilities of the two subscales. Alternatively, the estimates for \hat \sigma _S^2 and \hat \sigma _{SI,e}^2 from both analyses might be averaged and inserted into equation (2) with *k* = 5. Either way, the sampling error in the reliability estimate should be reduced by using all available data. We now purport to apply this method to an actual sample.

## Study 1

### Method

The final sample size of Study 1 included 395 female participants. Specifically, 859 students in a large Midwestern University (572 females, *M_Age_ =* 19.89, *SD* = 1.99) participated in this study as a part of a mass testing session. Their BMI was generally within the normal range (*M* = 22.4, *SD* = 3.34). Based on the cutoff criteria used by Fredrickson et al. (1998), 43 participants were overweight (BMI > 25.85) and 139 underweight (BMI < 20.80). This BMI distribution is similar to the one reported by Fredrickson and colleagues in their studies.

Participants answered via an online survey. Their answers on the SOQ were recorded even if they did not respect the instructions for completing the scale. Of these, only 585, 68% (395 female) completed the scale correctly. Errors usually involved attributing the same rank to several items. Given that objectification theory was formulated to address women’s experiences, we analyzed the female subsample (*n* = 395). ***Table 1*** reports the sample size, age, BMI, self-reported ethnicity, sexual orientation, and marital status for each of the studies.

**Table 1:** Sociodemographic characteristics for each of the studies.


	STUDY 1	STUDY 2	STUDY 3

*N*	395	93	195

Mean Age (*SD*)	19.88 (2.24)	37.76 (10.94)	34.29 (12.24)

Mean BMI (*SD*)	22.4 (3.34)	25.21 (5.36)	26.3 (7.75)

Ethnicity			

White	90%	83%	72%

Black	2%	3%	9%

Hispanic	2%	2%	5%

Asian	3%	9%	11%

Other ethnicity	3%	3%	3%

Sexual orientation			

Heterosexual	96%	89%	95%

Bisexual	2%	8%	5%

Other or no response	2%	3%	0%

Marital status			

Single	37%	30%	37%

In a committed relationship	59%	16%	21%

Married	3%	45%	30%

Divorced	1%	8%	10%

Widowed	0%	1%	2%


The SOQ was administered. Specifically, participants ranked the importance of five observable appearance-based attributes (weight, sex appeal, physical attractiveness, firm/sculpted muscles, measurements) and five non-observable competence-based attributes (physical coordination, health, strength, energy level, physical fitness) from 0 (*least impact*) to 9 (*greatest impact*). The difference between these ranks was taken as an indicator of self-objectification. The scores range from –25 to +25, with a higher score indicating more self-objectification.

Among the other measures collected, we included the Objectified Body Consciousness Scale (OBCS: McKinley & Hyde, 1996), a 24-item scale divided in three subscales: Body Surveillance (i.e., chronically thinking about how one appears to others), Body Shame (i.e., resulting from internalization of cultural body standards and failing to meet these standards) and Control Beliefs (i.e., believing that one can control one’s appearance with sufficient effort). Body surveillance and body shame are closely linked to self-objectification. These two concepts are commonly assessed in self-objectification studies as they, particularly body surveillance, have been theorized as manifestations of the self-objectification trait (for a review, see Moradi & Huang, 2008). OBCS refers to women’s experience of their body “as an object to be looked at” (McKinley & Hyde, 1996, p. 182). The OBCS constitutes the main alternative to the self-objectification questionnaire although the control beliefs subscale is less theoretically relevant to Fredrickson and Roberts’ formulation of objectification theory. The OBCS contains 24 items such as “During the day, I think about how I look many times” (body surveillance), “When I can’t control my weight, I feel like something must be wrong with me” (body shame), “I think a person can look pretty much how they want to if they are willing to work at it” (control beliefs). In this study, participants could report their answers on a scale from 1 (*strongly disagree*) to 7 (*strongly disagree*). Reliabilities for each subscale were relatively high: α_Surveillance_ = .84, α_Bodyshame_ = .86, α_Controlbeliefs_ = .76. This allows us to discard the possibility that participants were particularly careless in their answers (i.e., that they answered randomly, which could potentially explain the low reliability of the SOQ).

## Results and Discussion

### Descriptive statistics

The mean score on the SOQ was .91, *SD* = 12.77, which is comparable to the value obtained by Fredrickson et al. (1998) (*M* = 1.09, *SD =* 14.42). To assess the reliability of the scale, we first followed the recommendations of Hill and Fisher (2008) and calculated the correlation between the sums of scores on the appearance-based and competence-based items, using Spearman’s *rho*, which is appropriate for ordinal data. As expected, we found a correlation of –1 between rankings for the two types of attributes. Second, we evaluated the correlations between all items of the scale. Positive correlations between items that are in the same subscale and negative correlations between items that are not would be indicative of good reliability.

***Table 2*** shows that the direction of correlations was generally consistent with the model, but not always significant. With respect to the “competence” subscale, we see that out of 10 inter-item correlations, only four are positive and significant at the .05 level. For the appearance items, four out of ten correlations reach significance in the expected directions. Note however that we find one negative correlation between items belonging to the same subscale: “having firm/sculpted muscles” is negatively correlated with weight.

**Table 2:** Spearman inter-item correlations.


ITEM	1	2	4	7	9	3	5	6	8	10

Competence subscale										

1. Physical coordination	**1**									

2. Health	.13**	**1**								

4. Strength	**.08**	**.07**	**1**							

7. Energy level	.16**	.27**	**.05**	**1**						

9. Physical fitness	**.02**	**.07**	.10*	**.04**	**1**					

Appearance subscale										

3. Weight	–.27**	–.18**	–.23**	–.33**	–.26**	**1**				

5. Sex appeal	–.22**	–.47**	–.21**	–.33**	–.37**	**.03**	**1**			

6. Physical attractiveness	–.28**	–.48**	–.36**	–.35**	–.34**	.13**	.55**	**1**		

8. Firm/sculpted muscles	–.30**	–.21**	.02	–.24**	.02	–.12**	**.01**	**.07**	**1**	

10. Measurements	–.31**	–.29**	–.37**	–.30**	–.15**	.20**	**.04**	.11*	**–.05**	**1**


*Note*: **: *p* < .01 *: *p* < .05. Correlations involving items belonging to the same subscale are presented in **bold**.

In contrast, all correlations between items belonging to different subscales are negative and mostly significant. Given that items have to be ranked, it is not surprising that correlations across the two scales provide more consistent results than correlations within subscales. Specifically, when subjects are forced to rank several items, many patterns among the correlations are possible. The simplest pattern of a constant correlation would require that constant correlation to be small (when there are 10 items) and negative. In other words, the structure of the scale facilitates that items are negatively correlated, given that ranking an item at a high level limits the number of high level positions another item can take. Thus, it is not surprising that the negative correlation between appearance-competence pairs has higher magnitude than the positive correlations within subscales.

### Variance components

Using the lmer package (Bates et al., 2015) in R, we estimated restricted maximum likelihood (REML) of variance components for each of the two subscales. The results are displayed in ***Table 3***. Averaging the two reliabilities, we obtain an estimate of .31, which is very low: Typically, a value of Cronbach’s alpha below .70 is considered unsatisfactory (cf. Nunnally, 1978).

**Table 3:** REML estimates of variance components (and reliability) for the two subclasses of the Self-Objectification Questionnaire.


	Appearance	Competence

Random effect	Variance	Variance

Subject	.50	.51

Item	2.64	2.59

Error (Subject X Item)	5.67	5.63

Reliability	.30	.31


### Item by item analysis

Next, we assessed the contribution of each item by examining item-score correlations and changes in the reliability coefficient when an item is dropped. These results are displayed in ***Table 4***. As can be seen from this table, an item (“having firm/sculpted muscles”) from the appearance subscale seems clearly problematic. It is weakly positively correlated with other items in its own subscale and only moderately negatively correlated with the other subscale. Also dropping this item increases the reliability from .30 to .43.

**Table 4:** Item by Item analysis of the two subclasses.


	Reliability if item dropped	Correlation with score for subscale	Correlation with score for other subscale

Competence subscale			

1. Physical coordination	.28	.53	–.52

2. Health	.21	.59	–.59

4. Strength	.29	.45	–.45

7. Energy Level	.20	.58	–.58

9. Physical fitness	.34	.42	–.42

Appearance subscale			

3. Weight	.31	.48	–.47

5. Sex Appeal	.15	.61	–.61

6. Physical Attractiveness	.06	.66	–.65

8. Firm/Sculpted Muscles	.43	.31	–.31

10. Measurements	.28	.53	–.53


### Alternative subscales

Another way to assess the information contained within the SOQ items involves examining the reliability of each possible combination of two 5-item subscales from the 10 items provided. Ideally the combination proposed by Fredrickson et al. should maximize reliability. We performed this analysis by relying on the R statistical software.

Of the 252 possible combinations, the two subscales advocated by Fredrickson and Roberts (i.e., items 1, 2, 4, 7, 9 & 3, 5, 6, 8 and 10, respectively) yielded the highest alphas. The third best combination (2, 4, 7, 8, 9) only yielded an alpha of .006. With this exception, the distribution of these alphas was consistent with a normal distribution (*M* = –1.15, *SD* = .58, kurtosis = –.18, skewness = –.13). Most values were negative: When failing to reverse items, it is indeed possible to obtain negative alphas.

In this first set of computations, we did not reverse the direction of the items. In a second set of computations we allowed such a reversal. The “alpha” function from the psych package in R (Revelle, 2014) computed for each set of five items the best possible alpha taking into account the possibility of reversal. ***Table 5*** reports the 4 best series and their counterpart. Doing this generated a distribution with a maximum alpha of .67. It is pertinent to note that their counterpart did not have as high reliabilities, but these were still much higher than the .31 obtained without reversal.

**Table 5:** Best possible alpha for a series of 5 items.


	Items	Alpha

Series 1	Health, **Sex Appeal (R), Physical Attractiveness (R)**, Energy, Fitness	.67

Counterpart of series 1	Physical coordination (R), **Weight**, Strength (R), **Muscles, Measurements**	.46

Series 2	Health, Strength, **Sex Appeal (R), Physical Attractiveness (R)**, Energy	.67

Counterpart of series 2	Physical coordination (R), **Weight, Muscles**, Physical fitness (R), **Measurements**	.42

Series 3	Health (R), **Sex Appeal, Physical Attractiveness**, Energy (R), **Measurements**	.67

Counterpart of series 3	Physical coordination, **Weight** (R), Strength, **Muscles (R)**, Physical fitness	.38

Series 4	Physical Coordination, Health, **Sex Appeal (R), Physical Attractiveness (R)**, Energy	.67

Counterpart of series 4	**Weight**, Strength (R), **Muscles**, Physical fitness (R), **Measurements**	.38


*Note*: Appearance items are presented in **bold letters** and reversed items are followed by an “R”.

This analysis provides several insights. First, it *is* possible to achieve higher reliabilities if one allows reversal of items within the subsets. This means that significant associations between the two sub-dimensions exist in the original scale, at least when respondents are forced to rank instead of rate. Second, we notice that for each of the “best” combinations, items that are in the same direction belong to the same subscale. Third, we find that four items are present in all of the four “winners”: These items are sex-appeal, physical attractiveness, health and energy level. Fourth, the item “having firm/sculpted muscles” is the only one that features in none of the best combinations which is not surprising in view of its low correlation with the other items belonging to the same subscale.

### Convergent validity

One of the concerns that guided Noll and Fredrickson in the design of the SOQ was to develop a scale that would be independent of BMI (in line with objectification theory: Fredrickson & Roberts, 2007). Scores on the SOQ obtained in our sample fulfill this criterion: *r* = –.004, *ns*.

In spite of the closeness of their underlying constructs, the SOQ subscales were only weakly correlated with the three components of the OBCS (Pearson *r*s with the appearance subscale = .27 [*p* < .01], .08 [*p* = .11] and –.02 [*p* = .74] for body surveillance, body shame and control beliefs, respectively). Of course, correlations with the competence subscale are identical with the exception of a reversed sign.

We also examined the correlations between the four scores based on the highest reliabilities allowing for reversal of items and the three components of the OBCS. These correlations ranged from .13 (series 1) to .17 (series 4) for the three sub-dimensions of the OBCS. Correlations with the two other components were all smaller than .05.

To our knowledge, this is the first study to explicitly examine the reliabilities of the SOQ, using a range of analytic approaches that are suited to its rank order response format. While the inter-item correlations were mostly significant and in the expected directions, the alpha for the SOQ was quite low and inadequate by most contemporary standards. It is possible to increase alpha by including different combinations of items than the items that were originally formulated to go together, including reversing items from the appearance scale and including it with competence items (and vice versa). The “having firm/sculpted muscles” item, in particular seems problematic and it is possible that people are focusing on muscles as an indicator of physical strength, rather than focusing on the firm/sculpted nature of muscles as an indicator of physical appearance. With respect to convergent validity, the only significant correlation to emerge for the original SOQ was the relation between the SOQ and the Body Surveillance subscale of the OBCS. It is surprising that a stronger relation between the SOQ and this scale did not emerge, given the conceptual linkages between the constructs these measures are intended to assess.

## Study 2

Using generalizability theory, we attempted to provide an index of the reliability of the SOQ. Surprisingly, this reliability proved extremely low, suggesting that the scale does not tap a single construct. Recognizing the strength of the measure at a conceptual level (i.e., the measures maps onto Fredrickson and Roberts’ original notion of self-objectification) and at an empirical level (e.g., with expected relations between the SOQ and related predictors and outcomes, see Calogero, 2011; Moradi & Huang, 2008), we sought ways to improve the SOQ in a second study. In order to provide a more reliable index of self-objectification, we ran another study including two versions of the questionnaire: the original one (ranking response format) and another based on Likert scales (rating response format).

### Method

The final sample size of Study 2 contained 93 female participants recruited on MTurk. ***Table 1*** reports all sociodemographic characteristics.

First, participants were told the following: “The questions below identify 10 different body attributes. We would like you to indicate the extent to which each of these body attributes has an impact on your physical self-concept. For each attribute, please indicate whether the attribute has an extremely low or extremely high impact on your physical self-concept.” Participants rated each attribute on a visual analog scale from 1 (*Low impact*) to 11 (*High impact*). The full Likert scale of the Self-Objectification Questionnaire (LSOQ) is available in the appendix.

Second, the original SOQ was administered in such a way that participants could not attribute the same rank to two items. This controlled feature allowed all participants to correctly complete the entire scale. The order of administration of the two versions was counterbalanced. We also administered the OBCS (using scales from 1 to 6) and assessed the same sociodemographic questions as in Study 1. We also included two attention checks to ensure that participants paid attention to the items (Aust, Diedenhofen, Ullrich, & Musch, 2013). Seven participants were excluded for this reason. We also asked participants the following question: “Are there any reasons why you think we should not completely trust the data you provided? (e.g., you were interrupted, you did not read the instructions carefully, or you answered carelessly).” None replied positively.

## Results and Discussion

We computed Cronbach’s alphas on the two subscales of the original SOQ. Again, these were very low, replicating the findings of study 1 (.43 for the appearance items and .45 for the competence items). We submitted the Likert scale to a principal component analysis using the principal function from the psych package (version 1.6.8) in R (Revelle, 2015). To determine the number of components, we used the “very simple structure” criterion (Revelle & Rocklin, 1979), which evaluates the fit of the data compared to a model in which each item loads on a given number of components. Fixing this number at one (we had no reason to expect that items would load on multiple components), the best solution involved two components. The first component explained 31% of the total variance and the second the second 24%. As can be seen from ***Table 6***, “physical coordination”, “strength”, “energy level (stamina)”, “physical fitness”, and “health” had the highest loadings (> .65) on the first component and low loadings on the second (< .20). “Physical attractiveness”, “measurements (e.g., chest, waist, hips)”, “sex appeal”, “weight, and “firm/sculpted muscles” loaded on the second component. All but the muscles item had loadings equal or below .30 (in absolute values) on the first factor (with sex appeal and physical attractiveness having negative loadings).

**Table 6:** Loadings of the items on the Self-Objectification Questionnaire.


	Principal Component 1	Principal Component 2

Physical Coordination	**.76**	.00

Strength	**.78**	.11

Energy Level	**.77**	.03

Physical Fitness	**.70**	.19

Health	**.66**	–.13

Physical Attractiveness	–.19	**.84**

Sex Appeal	–.30	**.67**

Measurements	.18	**.77**

Weight	.19	**.61**

Firm/sculpted Muscles	.39	**.53**

Eigen value	3.13	2.45

% Variance explained	31	24


We averaged the two subscales separately. For the appearance items, alpha = .71 when including the “having firm/sculpted muscles” item and alpha = .72 when removing it. For the competence items, alpha = .80. As shown in ***Table 7***, the correlations between the two subscales were low (.14 or .03 depending on whether the muscles item was included or not).

**Table 7:** Pearson correlations between Likert-based subscales and self-objectification.


	1	2	3	4	5	6	7	8

1. SOQ (original scale)	–1.00 (14.71)							

2. Competence (Likert-scale)	–.46**	7.53 (1.80)						

3. Appearance (Likert-scale)	.45**	0.14	7.21 (1.71)					

4. Appearance (without muscles) (Likert-scale)	.51**	0.03	0.95**	7.55 (1.77)				

5. OBCS surveillance	0.50**	–.29**	.41**	.48**	3.65 (1.10)			

6. OBCS shame	.29**	–.20	.23**	.28**	.47**	3.06 (1.14)		

7. OBCS control	.00	–.05	.19*	.18*	.15	–.22*	4.44 (.82)	

8. BMI	–.11	–.07	–.18	–.10	–.06	.19	–.15	24.84 (5.92)

Cronbach’s alpha	.45	.79	.71	.72	.91	.89	.83	–


*Note*: **: *p* < .001 *: *p* < .05. means and standard deviations (in parentheses) are included in the diagonal.

As expected however, the competence subscale was correlated negatively with the original SOQ score and positively with its modified version whereas the reverse held for the appearance subscale. With respect to OBCS, each of the subscales had excellent reliability (see last row of ***Table 7***). The body surveillance subscale had a stronger positive correlation with the original SOQ (.50 instead of .27 in study 1) as well as with the appearance subscale of our modified version. Similarly, the body shame subscale had a positive correlation with the SOQ (.29 instead of .08) and our modified appearance subscale. By contrast, the competence subscale was weakly but negatively correlated with the shame and surveillance subscales. The control subscale of the OBCS bore no reliable relationship with any of the other scales except body shame.

The difference between the correlations involving the SOQ and the body shame and body surveillance subscales of the OBCS should be considered with caution. While the stronger correlations found in study 2 may be viewed as confirming the validity of the SOQ, it is important to note that correlations are extremely unstable before reaching high sample size (i.e., 250: Schönbrodt & Perugini, 2013). Note also that a comparison between the correlations obtained in the two studies allows to discard the null hypothesis that the two correlations are identical for the SOQ/surveillance correlation (Fischer’s Z = 2.33, *p* = .02) but not for the SOQ/shame correlation (Z = 1.87, *p* = .06).

Study 2 offers further evidence that Noll and Fredrickson’s original scale displays a poor reliability. This means that when women *have to* choose what aspects matter in their physical self-concept, the opposition between the “competence” and “appearance” does not seem to underlie their choices. When participants are *free* to provide their judgments on each item, we do indeed find that the “competence” and “appearance” items form two distinct components. This suggests that competence and appearance may indeed be separate concerns, but that valuing one does not entail neglecting the other. Finally, the present study confirms that the “having firm/sculpted muscles” item does not correspond clearly to one of the two aspects of self-objectification.

Note that, with respect to convergent validity, the relatively low sample size used in study 2, makes it difficult to draw firm conclusions as to the superiority of the adapted vs. original version of the SOQ. This issue, and validity in general, should be further explored in further studies.

Although we demonstrated that changing the measurement method can provide more accurate results, relying on Principal Component Analysis (PCA) is not without limitations. The disadvantage of a PCA is that this method is based solely on correlations among items, which produce factors that are hard to interpret. PCA therefore does not cover maximum variance, which can lead to information loss. Study 3 overcomes these limitations by testing the general structure of our Likert adaptation of the SOQ by relying on Factor Analysis.

## Study 3

The most appropriate alternative to PCA is Confirmatory Factor Analysis (CFA) because it allows to better capture the variance in variables. While PCA is a linear combination of variables, CFA is a measurement model that estimates latent variables, as opposed to an observed variable, is a variable that is not directly observed but is rather inferred from other variables that are observed. In this context, CFA is a structural equation modeling technique that decomposes the variance in the covariances between the items, the loadings of each item onto the factor, the covariances between the factors, and the variances of the factors. Consequently, Study 3 aims at analyzing the general structure of our Likert version of the SOQ in order to compare it with several concurrent models, using multiple fit indices to provide a multifaceted assessment of the models (Tanaka, 1993).

### Method

#### Sample size determination

**Figure 1:**
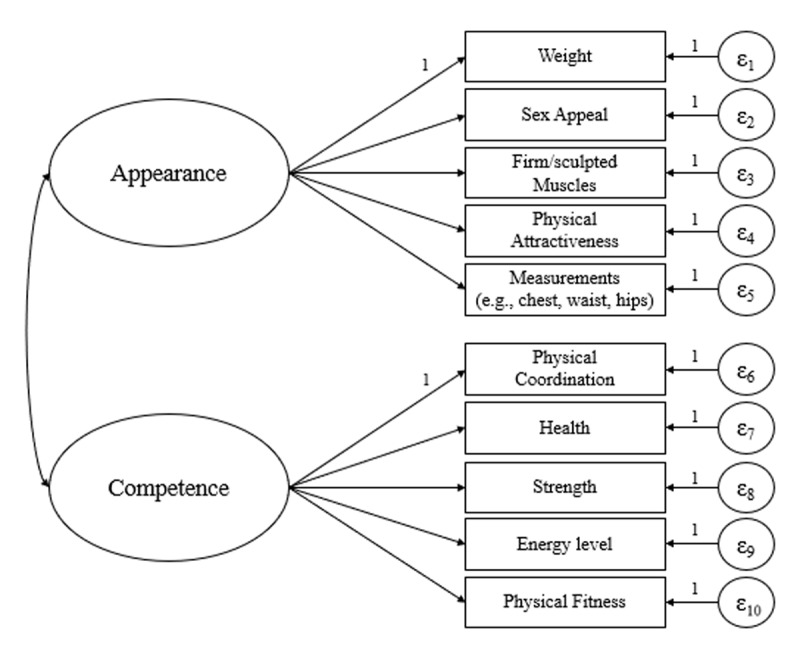
Structural Equation Modeling: Confirmatory Factor Analysis.

***Figure 1*** represents the general hypothesized model. Following the practical recommendation on sample size for structural equation modeling made by Kenny (2015), a 5 to 1 ratio of sample size to the number of free parameters is suggested (Bentler & Chou, 1987). The total number of elements in the initial covariance matrix is *k*(*k*+1)/2, where *k* equals the number of observed variables in the matrix. In this case, the total number of parameters = (10*11)/2 = 55. The parameters that have to be estimated in the model contain 10 observed variable variances (i.e., the attributes), 8 factor loadings (the unconstrained correlations between items and their latent construct), 2 latent variable variances (i.e., appearance and competence) and 1 latent variable covariance (i.e., covariance between both latent variables), which result in 21 free parameters. Thus, the minimum sample size equals to 5 (ratio)*21 (number of free parameters) = 105.

The final sample size of Study 3 contains 195 female participants recruited on MTurk. ***Table 1*** reports all sociodemographic characteristics.

## Results and Discussion

To test the general structure of both sub-dimensions of SOQ, appearance and competence, we performed a confirmatory factor analysis (CFA). The model was based on maximum likelihood estimation (see ***Figure 1***). Additionally, we examined the correlations between the residuals of items loading on the same scale and included the relevant error covariances when necessary. This model has a satisfactory model fit (see Model 2 in ***Table 8***). Importantly, all standardized factor loadings were significant and above the conventional threshold (>= .40) for competence (Physical coordination = .62, Health = .51, Strength = .71, Energy level = .57, Physical fitness = .86) and appearance (Weight = .76, Sex appeal = .67, Physical attractiveness = .67, Measurements = .63, Firm/Sculpted muscles = .49). This confirmed that all items exhibited sufficient loadings for both dimensions. Ultimately, the internal consistency was good for appearance (.77) and competence (.80).

**Table 8:** Comparisons of concurrent models.


	*χ^2^*	*DF*	*FI*	*RMSEA*

Model 1 – Single factor	152.930	31	.824	.142

**Model 2 – Two factors (*Figure 1*)**	**99.334**	**30**	**.900**	**.109**

Model 3 – Series 1	150.778	30	.826	.144

Model 4 – Series 2	149.000	30	.828	.143

Model 5 – Series 3	152.348	30	.824	.145

Model 6 – Series 4	149.860	30	.827	.143

Model 7 – Model 2 with muscles in the competence dimension	60.003	30	.957	.072

Model 8 – Model 2 without muscles	42.779	22	.966	.070


Next, we tested and compared several concurrent models. Model fit statistics for each model are reported in ***Table 8***. First, we created a model with a single factor (see Model 1). The very poor model fit statistics of Model 1 suggest that a single dimension using all items should not be considered. Second, Model 2 is the two factors general model described in the above paragraph and depicted in ***Figure 1***, demonstrating satisfactory model fit statistics. Third, we tested four models representing the different series of combination sets introduced in Study 1. All of them demonstrated poorer model fit statistics. Fourth, we tested a two factors model in which the item “having firm/sculpted muscles” was associated with the competence latent construct. This Model 7 demonstrated good model fit statistics. Finally, an additional two factors model was tested without the item “having firm/sculpted muscles” which led to an excellent fit (see Model 8 in ***Table 8***).

In sum, the results of Study 3 confirmed our previous findings and suggest that appearance and competence are two related but distinct dimensions. Moreover, rearranging the order of these items significantly deteriorated the model fit, suggesting that these alternative ways of considering these two dimensions are statistically (and conceptually) problematic. However, although the main model including the 10 attributes showed satisfactory model fit statistics, moving the item “having firm/sculpted muscles” to the competence dimension significantly increases the model fit, suggesting that this item relies more on the competence dimension as compared to the appearance dimension. Ultimately, removing this item from the whole scale provided a much better fit to the data.

## General Discussion

The purpose that guided this investigation was to propose a solution to evaluate the reliability of the self-objectification questionnaire (SOQ) developed by Noll and Fredrickson (1998) and updated by Fredrickson et al. (1998). We suggested to average the Cronbach’s alphas for the appearance and competence items separately. Surprisingly, applying this method to actual data yielded poor reliability and poor validity (i.e., weak correlation with OBCS) as well. However, two aspects of our data suggest that this instrument could be improved.

When computing the reliabilities of all possible combinations of five items, we found that the combination proposed by Fredrickson et al. (1998) yields the best reliability even if it remains poor. Second, if one allows items to be reverse scored, combinations of five items might produce a subscale with adequate reliability. In this study subscales producing reliabilities as high as .67, which is close to the generally accepted cut-off of adequate reliability, were observed. However, in Study 3, we found that such combinations of attributes demonstrated poorer model fit statistics as compared to a Likert rating implementation based on the original conceptualization of Noll and Fredrickson (1998).

We also noticed that the combinations of items offering the best reliabilities are consistent with the original distinction between competence and appearance items: Items that are in the same direction belong to the same subcategory of the SOQ. This suggests that ranking severely limits the reliability of the SOQ. If participants were allowed to freely choose the score they attribute to a given item without being constrained by the others, reliabilities may be much higher. This was indeed confirmed in Study 2 and Study 3, in which Likert scales were used.

Moreover, the three studies suggest that the “sculpted/firm muscles” item does not fit clearly in the appearance subscale. Muscles are both associated to competence (e.g., representing physical strength) and appearance (e.g., representing a firm or sculpted muscular appearance). Most importantly, Study 3 demonstrated that removing this item from the whole scale provided a much better fit to the data.

Besides its poor reliability, another problem posed by the SOQ is that a large proportion of participants (more than 30% in the sample of our first study) fail to respond to it properly. This may reflect lack of attention to the instructions that force participants to attribute different scores to each item and could be remedied for computerized data collection by forcing participants to provide a unique numeric response to each item (it would be substantially more difficult to address this issue when paper/pencil versions of the scale must be utilized). This may, however, also be related to the multidimensionality of the scale: Participants may not find it meaningful to rank order these items on a single dimension and to choose between two constructs that a healthy person may value highly. Thus, impelling participants to use the rank order response format may provide more correctly completed data, but by artificially constraining the degree to which they spontaneously think about their own physical appearance relative to their physical competence.

Finally, the original version of the SOQ only displayed a relatively low correlation with closely related constructs, including the body shame (especially in study 1) and body control subdimensions of objectified body consciousness (McKinely & Hyde, 1996). This should come as no surprise if one considers that reliability constrains validity. Conversely, however, the fact that the scale has been often and successfully used to predict outcomes of theoretical relevance and interest (cf. Calogero, 2011; Moradi & Huang, 2008) suggests that the relation between self-objectification and these outcomes is so strong that, behind the “noise” due to its poor reliability, it manages to capture a significant amount of the variance in these constructs. Yet, the interpretation of studies that have revealed null findings between hypothesized predictors or outcomes of the SOQ should be interpreted somewhat with caution, given that these null findings could be explained, at least in part, due to the low reliability of the SOQ. Note that, in Study 2, we found that only the appearance subscale (in its Likert version) was strongly associated with the two components of the OBCS that are of theoretical interest here *i.e*., shame and surveillance. This further justifies the consideration of the appearance and competence components as distinct.

In the same vein, while Study 1 demonstrated strong inter-item correlations between the two dimensions of the original instrument, this is not surprising as the rank order method forces respondents to oppose these two dimensions, which consequently leads to stronger (negative) associations between these sets of items. As shown in Study 2, when using the Likert version of the SOQ in which participants are not forced, the “appearance” and “competence’ dimensions are only weakly correlated. The remarkable finding that the “competence” subdimension is weakly associated to “appearance” and other measures related to self-objectification suggests that there is no necessary tradeoff between the competence aspect on the one hand and body shame/surveillance on the other hand. It invites scholars to consider how these two dimensions can be articulated in women’s physical self-concept: Some women may attach great importance to both aspects while others’ self-concept may be quite independent of them.

Results of the three studies lead us to discourage the use of the original ranking format of the SOQ and rely on the Likert version of the Self-Objectification Questionnaire (LSOQ, see appendix). In this way, the conceptual strengths of the SOQ (e.g., it is a face valid measure of self-objectification as conceptualized by Fredrickson and Roberts, 1997) can be retained while the psychometric properties of the scale can be improved.

## Data Accessibility Statement

The data, codes and questionnaires used in this study are available here: *https://osf.io/na48u/*.

## Footnotes

We use the term “subject” rather than “participant” to avoid confusions given the use of this term in the psychometric literature and in statistical terminology.

## Appendix

**Likert version of the Self-Objectification Questionnaire (LSOQ)**.

The questions below identify 10 different body attributes. We would like you to indicate the extent to which each of these body attributes has an impact on your physical self-concept. For each attribute, please indicate whether the attribute has an extremely low or extremely high impact on your physical self-concept.

From 1 (*Low impact*) to 11 (*High impact*)

1. Health
2. Weight
3. Strength
4. Sex appeal
5. Physical attractiveness
6. Energy level (e.g., stamina)
7. Having firm/sculpted muscles
8. Physical fitness
9. Measurements (e.g., chest, waist, hips)
10. Physical coordination

Appearance score = average of the following items: 2, 4, 5, 7, 9.

Competence score = average of the following items: 1, 3, 6, 8, 10.
